# US Emergency Department Visits and Hospital Discharges Among Uninsured Patients Before and After Implementation of the Affordable Care Act

**DOI:** 10.1001/jamanetworkopen.2019.2662

**Published:** 2019-04-19

**Authors:** Adam J. Singer, Henry C. Thode, Jesse M. Pines

**Affiliations:** 1 Department of Emergency Medicine, Renaissance School of Medicine, Stony Brook University, Stony Brook, New York; 2 US Acute Care Solutions, Canton, Ohio

## Abstract

**Importance:**

The US Patient Protection and Affordable Care Act of 2010 (ACA) was enacted in 2010 with several provisions that targeted reducing numbers of uninsured Americans.

**Objective:**

To assess the numbers and proportion of emergency department (ED) visits (2006-2016) and hospital discharges (2006-2016) by uninsured patients, focusing on the 2014 ACA insurance reforms (Medicaid expansion, individual mandate, and private insurance exchanges).

**Design, Setting, and Participants:**

Cross-sectional study of visitors to US EDs and patients discharged from US hospitals using National Hospital Ambulatory Care Survey data and Healthcare Cost and Utilization Project data, respectively, from 2006 to 2016. Data analysis took place in February 2019.

**Main Outcomes and Measures:**

Numbers and proportions of total and uninsured ED visits and hospital discharges. Simple descriptive statistics and interrupted time-series analysis were used to assess changes in uninsured visits over time and after the implementation of insurance provisions in 2014.

**Results:**

There were an estimated 1.4 billion US ED visits from 2006 to 2016 and 405 million hospital discharges from 2006 to 2016. Over the study period, ED visits increased by 2.3 million per year, while hospital discharges decreased from approximately 38 million per year prior to 2009 to approximately 36 million per year after, with no clear decrease after 2013. Proportions of uninsured ED visits were largely unchanged from 2006 (16%) until 2013 (14%) (−0.2 percentage point per year; 95% CI, −0.46 to −0.01 percentage point; *P* = .11) but then decreased by 2.1 percentage points per year from 2014 to 2016 (95% CI, −4.3 to −1.8 percentage points; *P* = .003), with uninsured visits composing 8% of the total in 2016. For patients aged 18 to 64 years, uninsured ED visits declined from approximately 20% from 2006 through 2013 to 11% in 2016 (3.1% decrease per year after 2013; 95% CI, −4.3 to −1.8 percentage points; *P* = .003). The proportion of hospital discharges by uninsured patients remained steady at approximately 6% from 2006 to 2013, then declined to 5% in 2014 and 4% in 2016. Similar changes were seen for patients aged 18 to 64 years, with a decrease in hospital discharges from 10% to 7% over the study period.

**Conclusions and Relevance:**

Proportions of ED visits and hospital discharges by uninsured patients decreased considerably after the implementation of the 2014 ACA insurance provisions. Despite these changes, approximately 1 in 10 ED visits and 1 in 20 hospital discharges were made by uninsured individuals in 2014 to 2016. This suggests that continued attention is needed to address the lack of insurance in US hospital visits, particularly among people aged 18 to 64 years who have less access to government-sponsored insurance.

## Key Points

**Question:**

Were the 2014 insurance provisions in the Affordable Care Act (ACA) associated with changes in US emergency department (ED) visits or hospital discharges among uninsured individuals?

**Findings:**

In this cross-sectional study of 1.4 billion US ED visits from between 2006 and 2016 and 405 million hospital discharges between 2006 and 2016, proportions of ED visits and hospital discharges by uninsured patients declined from 16% to 8% and 6% to 4%, respectively, after the 2014 ACA insurance expansions. Among patients aged 18 to 64 years, declines were from 20% to 11% and 10% to 7%, respectively.

**Meaning:**

The implementation of the ACA insurance provisions in 2014 was associated with decreased ED and hospital use by uninsured individuals, but by 2016 many patients seeking care in US hospitals remained uninsured.

## Teaser

This cross-sectional study uses data from the US National Hospital Ambulatory Care Survey and the Healthcare Cost and Utilization Project to evaluate the association between implementation of the Affordable Care Act (ACA) and rates of emergency department (ED) visits and hospitalizations by uninsured individuals.

## Introduction

One of the main goals of the Patient Protection and Affordable Care Act of 2010 (ACA) was to reduce the number of people without health insurance in the United States while also improving health care quality and reducing cost.^1^ Whether these changes have achieved their goals remains to be seen. Several ACA provisions targeted health insurance: (1) expanding Medicaid eligibility, which later became optional for states (2014); (2) the creation of health insurance exchanges (2014); (3) regulations on health plans that allowed young adults to remain on their parents’ insurance until age 26 years and disallowed excluding or charging higher rates for patients with preexisting conditions (2010); (4) requirements that most individuals have health insurance (implemented 2014 but repealed in 2018); and (5) penalties to employers for not offering coverage for employees (2016). In addition, there were other ways the ACA might affect coverage, such as limits on experience rating and subsidies. To our knowledge, no reports have presented nationally representative data describing the longitudinal associations of the ACA with both ED visits and hospital discharges together.

We examined trends in ED visits and hospitalizations over the decade from 2006 to 2016, including visit rates and changes in insurance coverage, with a focus on visits after 2013, when the ACA insurance provisions of Medicaid eligibility expansion, health insurance exchanges, and the individual mandate^2^ went into effect. Specifically, we aimed to answer the following question: was ACA expansion in 2014 associated with significant changes in the number of ED visits and hospitalizations in the US overall and by uninsured individuals in particular?

## Methods

### Study Design and Setting

We conducted a retrospective secondary analysis of data from the National Hospital Ambulatory Care Survey (NHAMCS) (2006-2016) and the Healthcare Cost and Utilization Project (HCUP) (2006-2016) from the National Inpatient Sample, and followed the Strengthening the Reporting of Observational Studies in Epidemiology (STROBE) reporting guideline for cross-sectional studies. Survey data from NHAMCS include a nationally representative sample of US ED visits, and HCUP is the largest collection of longitudinal hospital data. Because the data are publicly available and deidentified, the institutional review board of Stony Brook University does not consider this to be human subjects research, and it is therefore exempt from review.

### National Hospital Ambulatory Care Survey 

Data from the NHAMCS include a nationally representative sample of visits to hospital-based EDs. The NHAMCS is conducted by the Centers for Disease Control and Prevention’s National Center for Health Statistics. In the ED portion of the survey, a rolling sample of approximately 400 hospital EDs provide data on a random sample of patient visits annually. The range of hospitals sampled in the survey comprises noninstitutional general and short-stay hospitals (excluding federal, military, and Veterans Health Administration hospitals) located in the United States. The weighted 4-stage probability sample allows for extrapolation of national estimates from primary sampling units in all 50 states and the District of Columbia. The US Census Bureau acts as the data collection agent for the NHAMCS and trains hospital staff at each sampled ED to collect data, while field representatives review the case report forms to ensure data quality. A detailed description of the NHAMCS, including sample design, data collection procedures, field quality control, data processing, and estimation procedures, is available on the National Center for Health Statistics website.^3^

### Healthcare Cost and Utilization Project 

The data from the HCUP National Inpatient Sample were extracted from HCUPnet, the online tool available on the website of the Agency for Healthcare Research and Quality. The National Inpatient Sample has information on all hospital discharges, including data on demographic characteristics (age, sex, payer) as well as diagnosis and other variables from US community hospitals as a 20% stratified sample from the Agency for Healthcare Research and Quality’s State Inpatient Databases.

### Outcomes

Primary outcomes of interest were the number of ED visits, hospital admissions from the ED, and hospital discharges over the study period. Each outcome was examined by insurance type (uninsured, Medicaid, private insurance, and Medicare) both in terms of total numbers and relative payer mix expressed as a percentage of all patients. A planned subgroup analysis included a subset of patients aged 18 to 64 years, as this group has less access to government-sponsored insurance.

### Statistical Analysis

Binary data are presented as numbers and frequencies. Interrupted time series using Joinpoint^4^ software was used to determine whether the proportions of ED visits and discharges changed, especially in the uninsured category, from 2014 to 2016 compared with those in earlier years. Joinpoint models are linear regression models where lines with different slopes are connected together at join points, which are points where changes can be detected.^5^ Joinpoint software takes trend data and fits the simplest model that the data allow. The program was started with the minimum number of specified join points equal to 0 (ie, a straight line) and tested whether more join points must be added to the model to identify changes in trend that were statistically significant. The tests of significance are determined using a Monte Carlo permutation method, with 5000 replications with unautocorrelated errors, to identify models that are statistically different from a single linear regression line.^5^ The level of significance was set at a 2-sided *P* value of .05.

## Results

### Visits to the ED and Hospital Admissions Originating From the ED

**Figure 1.**
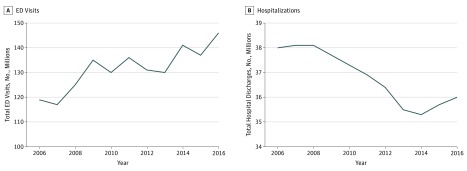
Annual US Emergency Department (ED) Visits and Hospitalizations, 2006 to 2016

**Figure 2.**
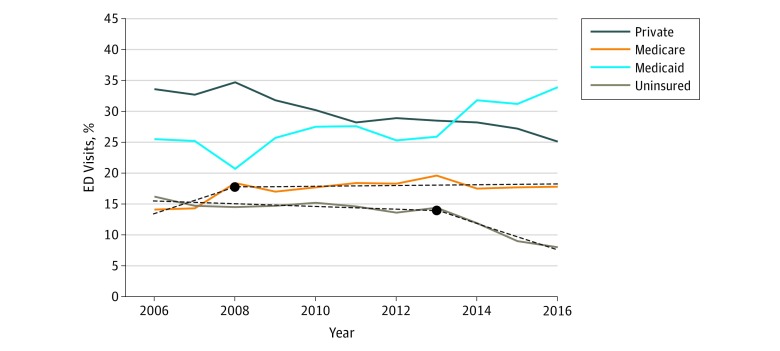
Percentages of US Emergency Department (ED) Visits by Insurance Type, 2006 to 2016 Solid lines denote actual data. Solid lines without superimposed dashed lines indicate that the best regression line was linear, ie, no change in slope. Dashed lines indicate models that had a significant change in slope at some time point. Year at which the slope changed is shown as a black circle.

From 2006 to 2016, there were an estimated 1.4 billion ED visits nationwide. The number of ED visits between 2013 and 2016 increased as expected based on previous time trends of 2.3 million per year (Figure 1A). During the same period, the proportion of ED visits by patients with Medicaid increased steadily, while there was an abrupt 2.1 percentage point (95% CI, −4.3 to −1.8 percentage points) per year decrease in visits by uninsured individuals after 2013 (Table), so that uninsured visits made up only 8% of visits in 2016. The percentages of annual ED visits by patients receiving Medicaid increased from 26% in 2013 to 34% in 2016. During the same period, visits by uninsured patients decreased from 14% to 8% (change per year, −2.1 percentage points; 95% CI, −4.3 to −1.8 percentage points; *P* = .003). Shift in payer mix after the implementation of the 2014 insurance provisions is demonstrated in Figure 2. There was a small but significant decrease in the percentage of patients with private insurance (−0.8 percentage point/year; 95% CI, −1.0 to −0.1 percentage point/year; *P* < .001). Medicare use increased from 2006 until 2008 and then leveled off.

**Table.** Trend Models for ED Visits and Hospitalizations

Dependent Variable	Model	Slope 1 (p^a^)	Join Point	Slope 2 (p^a^)	*P* Value^b^
Trend models for % of ED visits by insurance type					
% Private insurance visits	Linear	−0.8 (0.00003)	NA	NA	.81
% Medicare visits	1 Join point	2.1 (0.11)	2008	0.06 (0.63)	.02
% Medicaid visits	Linear	0.9 (0.004)	NA	NA	.18
% Uninsured visits	1 Join point	−0.2 (0.11)	2013	−2.1 (0.003)	.001
Trend models for No. of ED visits by insurance type					
Total No. of ED visits	Linear	2.3 (0.0005)	NA	NA	.49
No. of private insurance visits	Linear	−0.4 (0.07)	NA	NA	.30
No. of Medicare visits	1 Join point	3.4 (0.05)	2008	0.4 (0.02)	.02
No. of Medicaid visits	Linear	1.8 (0.0007)	NA	NA	.24
No. of uninsured visits	1 Join point	0.04 (0.86)	2013	−2.6 (0.03)	.005
Trend models for % of hospital discharges by insurance type					
% Private insurance visits	Linear	−0.5 (0.00008)	NA	NA	.30
% Medicare visits	Linear	0.3 (0.0006)	NA	NA	.56
% Medicaid visits	Linear	0.4 (0.0003)	NA	NA	.38
% Uninsured visits	1 Join point	−0.03 (0.61)	2013	−0.6 (0.04)	.02

Abbreviations: ED, emergency department; NA, not applicable.

^a^ The numbers in parentheses indicate the significance level of alternative hypothesis that the slope does not equal 0 vs null hypothesis that the slope is 0. Slopes indicate change per year in percentage points.

^b^ Significance of alternative hypothesis of 1 join point vs null hypothesis of 0 join points (straight line). Slopes indicate change per year in percentage points.

This pattern was also seen in the number of ED visits, where the number of uninsured ED visits showed no significant trend from 2006 (16%) through 2013 (14%) (−0.2 percentage point per year; 95% CI, −0.46 to −0.01 percentage point; *P* = .11). In contrast, the number of ED visits by uninsured individuals decreased by 2.6 million per year from 2013 to 2016 (95% CI, −4.4 million to −0.8 million; *P* = .03) (Table).

**Figure 3.**
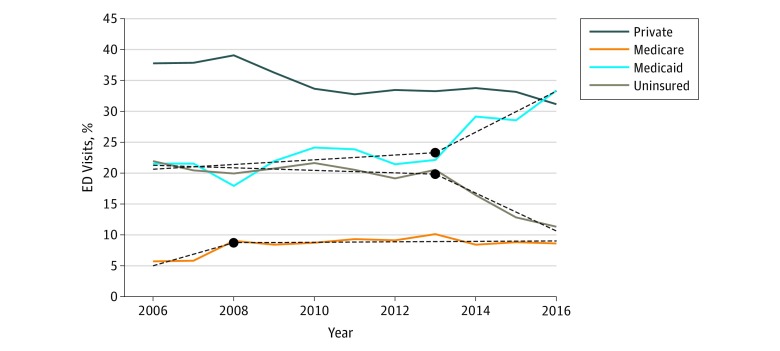
Percentages of US Emergency Department (ED) Visits by Insurance Type for Patients Aged 18 to 64 Years, 2006 to 2016 Solid lines denote actual data. Solid lines without superimposed dashed lines indicate that the best regression line was linear, ie, no change in slope. Dashed lines indicate models that had a significant change in slope at some time point. Year at which the slope changed is shown as a black circle.

The changes in ED visits after 2013 were similar but more pronounced in patients aged 18 to 64 years (Figure 3). There was a small increase in Medicaid use from 2006 to 2013, then an increase of 3.3 percentage points per year from 2013 to 2016 (95% CI, 0.4-6.2 percentage points; *P* = .04). There was a corresponding small decrease in uninsured patients from 2006 (20%) to 2013 (11%), and then a decrease of 3.1 percentage points per year from 2013 to 2016 (95% CI, −4.3 to −1.8 percentage points; *P* = .003). Medicaid use increased from 2006 to 2008 and then leveled off.

The number of ED visits resulting in hospital admission increased from 2006 to 2010, decreased until 2014, and showed a slight increase from 2014 to 2016. By payer group, the number of hospital admissions that originated from the ED generally decreased over the study period in all payer groups except Medicare. Medicare ED admissions increased from 2006 to 2009 by approximately 0.8 million per year and decreased by 0.4 million per year thereafter (95% CI, −0.6 million to −0.2 million; *P* = .01). Similar patterns by payer are seen when using percentage of ED admissions as the outcome variable.

### Hospital Discharges

**Figure 4.**
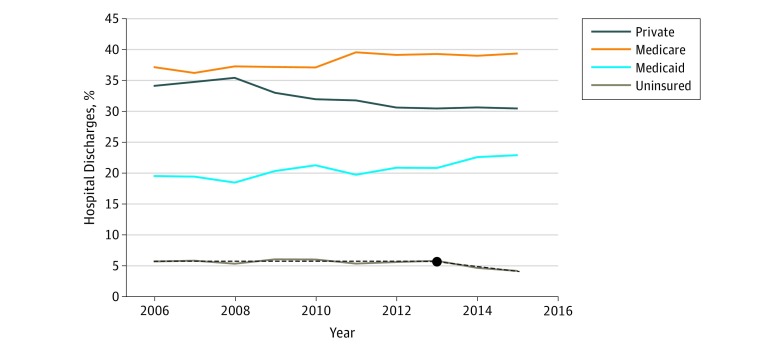
Percentages of US Hospital Discharges by Insurance Type, 2006 to 2016 Solid lines denote actual data. Solid lines without superimposed dashed lines indicate that the best regression line was linear, ie, no change in slope. Dashed line indicates model that had a significant change in slope at some time point. Year at which the slope changed is shown as a black circle.

Between 2006 and 2016, there were 405 million hospital discharges. Hospital discharges remained steady at approximately 38 million per year prior to 2009, decreased to approximately 36 million per year between 2009 and 2013, and then appeared to level off (Figure 1B). There was no clear decrease in the number of hospital discharges after 2014. However, when examining changes in payer mix, we observed similar changes as were seen with ED visits after the ACA insurance expansions, specifically by insurance type (Figure 4). The percentages of annual hospital discharges of uninsured individuals remained steady at approximately 6% from 2006 to 2013, then declined to 5% in 2014 and 4% in 2016 after ACA expansion (decrease per year, 0.6 percentage point; 95% CI, −1.00 to −0.01 percentage point; *P* = .04). Similar patterns were seen in patients aged 18 to 64 years, with a decrease from 10% to 7% over the study period.

## Discussion

The ACA has several provisions focused on improving health insurance coverage and reducing the number of uninsured Americans. Some of these provisions rolled out early in the ACA (ie, allowing people to stay on their parents’ insurance until age 26 years). However, more dramatic changes in insurance coverage were implemented in January 2014: the expansion of Medicaid in selected states, establishment of health insurance exchanges intended to create private (non–employer-based) markets for coverage, and the individual mandate. These changes resulted in extended coverage to 20 million previously uninsured individuals and a 21% increase in Medicaid enrollment.^6^ To date, 32 states and the District of Columbia have elected to expand coverage.

Our study is one of the few that have simultaneously examined ED visits and all hospital discharges. We demonstrate clear associations between ACA legislation and changes in ED visits and hospital discharges, as well as other broad trends in US health care policy. Visits to the ED increased rapidly until 2010, but then grew more slowly after that year, averaging 130 million to 140 million hospital-based visits per year, while the number of hospitalizations has been decreasing from the ED and overall during the same period. This demonstrates a continued importance of EDs in US medical care, despite efforts to direct patients to other settings. However, after 2012, there was a decrease in inpatient hospitalizations observed from the ED and overall. This may be due to an increased use of outpatient services, observation services (as opposed to inpatient services), and nonhospital care settings. These trends in hospitalizations are associated with ACA provisions such as new payment rules, payment models, promotion of patient-centered medical homes, and the expansion of quality measurement and also with the expansion of alternatives such as urgent care, freestanding emergency care centers, and telemedicine. Specifically, since the ACA rollout, EDs are increasingly discharging patients home after evaluation. Increased coverage and greater access to timely outpatient follow-up and medical homes may in part explain this trend. Alternatively, with changing rules regarding observation services, more ED patients may be observed in ED observation units that do not count as inpatient hospitalizations. A general culture shift and emphasis on value-based care after the ACA may have also contributed to decreased hospitalizations from the ED. Nevertheless, the combination of a continued increase in ED visits and decreasing hospitalizations despite increasing rates of insurance coverage suggests that ED visits may be less preventable than inpatient hospitalizations, which may be more discretionary.

After 2014, there was a significant change in the payer mix for ED visits and hospital discharges, with a proportional shift from uninsured individuals to those receiving Medicaid, as well as declines in private insurance. These changes were accentuated among adult patients aged 18 to 64 years, who are at the highest risk for being uninsured because many are not eligible for state-level coverage or Medicare. Findings were similar for hospitalizations, which showed similar reductions in uninsured visits. Prior studies^7,8,9,10,11,12,13,14^ have also demonstrated that Medicaid expansion in particular is associated with changes in insurance mix in EDs and hospitals; however, association between expansion and the numbers of people using the ED has not been demonstrated. Future work is needed in this area to further dissect the effects of these policies at the national level, on individual states, in specific populations of patients, and with respect to health outcomes. Additional work is needed to examine how these trends have affected out-of-pocket costs for patients and overall costs of care. Longer-term studies will also be helpful to determine the continued or delayed impact of insurance changes.

At the end of the study period (2016), we found that, despite decreasing rates of uninsured ED visits and hospitalizations, nearly 1 in 10 ED visits and 1 in 20 hospitalizations were still uninsured. This represents an important gap that policy makers should continue to address, as lack of insurance coverage is associated with worse health outcomes, and the United States is one of the only developed countries that does not guarantee health insurance coverage to all citizens.^15^

### Limitations

There are several limitations to this study. The first limitation is the descriptive nature of our work, which does not explicitly isolate the effects of the insurance expansions (ie, separating states by Medicaid expansion), but rather examines trends as a whole. It is possible that factors other than insurance expansions or the ACA may account for some of the trends reported. Another major limitation is the retrospective nature of the study and our reliance on publicly available hospital-based databases. These are limited because of the inability to account for repeat visits, or how visits evolved in other parts of the health care system, such as in outpatient clinics. In addition, because the databases contain a limited set of variables, we could not adjust for many confounding variables that may have affected ED visits and hospital discharges, such as changes in treatment patterns, reasons for visit, or other health policies that may have occurred concurrently. Also, our study only included 3 years after Medicaid expansions. Therefore, we cannot estimate or comment on more current and future trends.

## Conclusions

Based on nationally representative administrative databases, the percentage of ED visits and hospital discharges by uninsured patients decreased considerably after the ACA insurance expansions of 2014. This was balanced by increases in ED visits and hospital discharges by patients with Medicaid. Furthermore, these shifts in payer mix were especially prominent among individuals aged 18 to 64 years. Overall, ED visits did not appear to be associated with ACA expansions.
